# Nationwide population-based cohort study of adverse obstetric outcomes in pregnancies with myoma or following myomectomy: retrospective cohort study

**DOI:** 10.1186/s12884-020-03406-9

**Published:** 2020-11-23

**Authors:** Se Jin Lee, Hyun Sun Ko, Sunghun Na, Jin Young Bae, Won Joon Seong, Jong Woon Kim, Jaeeun Shin, Hae Joong Cho, Gyu Yeon Choi, Jinsil Kim, Geum Joon Cho, In Yang Park

**Affiliations:** 1 Department of Obstetrics and Gynecology, Kangwon National University, School of Medicine, Chuncheon, South Korea; 2 Department of Obstetrics and Gynecology, Seoul St. Mary’s Hospital, College of Medicine, The Catholic University of Korea, 222, Banpo-daero, Seocho-gu, Seoul, 06591 Republic of Korea; 3 Department of Obstetrics and Gynecology, School of Medicine, Catholic University of Daegu, Daegu, South Korea; 4 Department of Obstetrics and Gynecology, Kyungpook National University, School of Medicine, Daegu, South Korea; 5 Department of Obstetrics and Gynecology, Chonnam National University Medical School, Gwangju, South Korea; 6 Bucheon St. Mary’s Hospital, College of Medicine, The Catholic University of Korea, Bucheon, South Korea; 7 Department of Obstetrics and Gynecology, College of Medicine, Wonkwang University, Iksan, South Korea; 8 Department of Obstetrics and Gynecology, Soonchunhyang University Seoul Hospital, Seoul, South Korea; 9 Department of Obstetrics and Gynecology, Korea University Guro Hospital, Korea University College of Medicine, 148 Gurodong-ro, Guro-gu, Seoul, 08308 Republic of Korea

**Keywords:** Myoma, Myomectomy, Pregnancy outcome, Uterine rupture

## Abstract

**Background:**

Our objective was to evaluate risks of adverse obstetric outcomes in pregnancies with myoma(s) or in pregnancies following myomectomy.

**Methods:**

We analyzed the national health insurance database, which covers almost the entire Korean population, between 2004 and 2015. The risks of adverse pregnancy outcomes in pregnancies with myoma(s) or in pregnancies following myomectomy, compared to those in women without a diagnosed myoma, were analyzed in multivariate logistic regression analysis.

**Results:**

During the study period, 38,402 women with diagnosed myoma(s), 9890 women with a history of myomectomy, and 740,675 women without a diagnosed myoma gave birth. Women with a history of diagnosed myoma(s) and women with a history of myomectomy had significantly higher risks of cesarean section (aOR 1.13, 95% CI 1.1–1.16 and aOR 7.46, 95% CI 6.97–7.98, respectively) and placenta previa (aOR 1.41, 95% CI 1.29–1.54 and aOR 1.58, 95% CI 1.35–1.83, respectively), compared to women without a diagnosed myoma. And the risk of uterine rupture was significantly higher in women with previous myomectomy (aOR 12.78, 95% CI 6.5–25.13), compared to women without a diagnosed myoma, which was much increased (aOR 41.35, 95% CI 16.18–105.69) in nulliparous women. The incidence of uterine rupture was the highest at delivery within one year after myomectomy and decreased over time after myomectomy.

**Conclusions:**

Women with a history of myomectomy had significantly higher risks of cesarean section and placenta previa compared to women without a diagnosed myoma.

**Supplementary Information:**

The online version contains supplementary material available at 10.1186/s12884-020-03406-9.

## Background

Uterine myomas (leiomyomata, fibroids) are the most common tumor of the reproductive tract, with a prevalence of 20–25% [1] and a cumulative incidence of 70% in women of reproductive age [2].

It has been reported that the presence of fibroids is associated with infertility, spontaneous abortion, fetal malpresentation, placenta previa, preterm birth, cesarean section, and peripartum hemorrhage [3]. Although surgical interventions such as myomectomy have been tried in infertile women without specific causes, it is unclear whether the treatment of uterine fibroids can improve pregnancy outcomes, except for cavity-distorting myomas (submucosal, or intramural with a submucosal component) [4].

Because there is insufficient evidence that myomectomy improves pregnancy outcomes, academic societies including the American Society for Reproductive Medicine (ASRM) and the Society of Obstetricians and Gynaecologists of Canada (SOGC) are against myomectomy in asymptomatic women with non-cavity-distorting myomas [5, 6]. Nevertheless, in US Census Bureau population projections, it was estimated that myomectomies are predicted to increase 31% between 2007 and 2050 [7]. In Korea, the number of women who underwent myomectomy have increased 37.3% between 2006 and 2010 [8]. Women in their 30s and 40s, who are a major population for pregnancy, have been major candidates for myomectomy, although there have been poor evidences about obstetric outcomes in women with myoma and previous myomectomy [9].

The most serious concern in pregnancies after myomectomy is the risk of uterine rupture, which can result in significant increased morbidity and mortality for both the mother and the fetus. The incidence of uterine rupture after prior myomectomy has been reported to range from 0.2 to 3.7% in women with prior myomectomy [10].

The purpose of this study was to evaluate adverse pregnancy outcomes in women with a history of diagnosed myoma or myomectomy, including prevalence of uterine rupture in women with myomectomy, according to the time interval after myomectomy.

## Methods

### The characteristics of the dataset

The Korea National Health Insurance (KNHI) program covers 97% of Korean population. The KNHI claims database which contains these individuals’ information is centralized database that most of the information about the prevalence of different disease and procedures can be obtained except procedures not covered by insurance. As part of the KNHI system, children aged 4–80 months are eligible for a National Health Screening Program for Infants and Children (NHSP-IC) [11]. An NHSP-IC consists of seven consecutive health examinations based on age groupings. At ages 4–9, 9–18, 18–30, 30–42, 42–54, 54–66, and 66–80 months, health examinations are performed. An NHSP-IC is composed of history taking, physical examination, anthropometric examination, developmental screening, and visual acuity testing based on the child’s age. Women’s data in the KNHI claims database were connected to the data of their offspring contained within the NHSP-IC database.

ICD-10 codes were used for women’s health condition and obstetric diagnosis in KNHI claim database. ICD-10 codes for uterine myomas were D25. Surgery codes for myomectomy were JR4123, JR4124, JR4127, JR4128 and JR4129. To evaluate the effects of myomectomy according to types of myomas on pregnancy outcomes, myometomy groups were divided to submucosal (D25.0) or intramural (D25.1) myomas, and subserosal myomas (D25.2) or unspecified myomas (D25.9), by ICD codes.

Data on preterm birth and birth weight were corrected from the NHSP-IC database.

### Outcomes

We analyzed the KNHI claims database, between 2004 and 2015. Using the database, we identified all women who had had deliveries between January 1, 2014, and December 31, 2015. We also identified whether these women had a diagnosis of uterine myoma based on ICD-10 codes before pregnancy and whether these women had a myomectomy using procedure codes from the Health Insurance Medical Care Expenses.

**Fig. 1:**
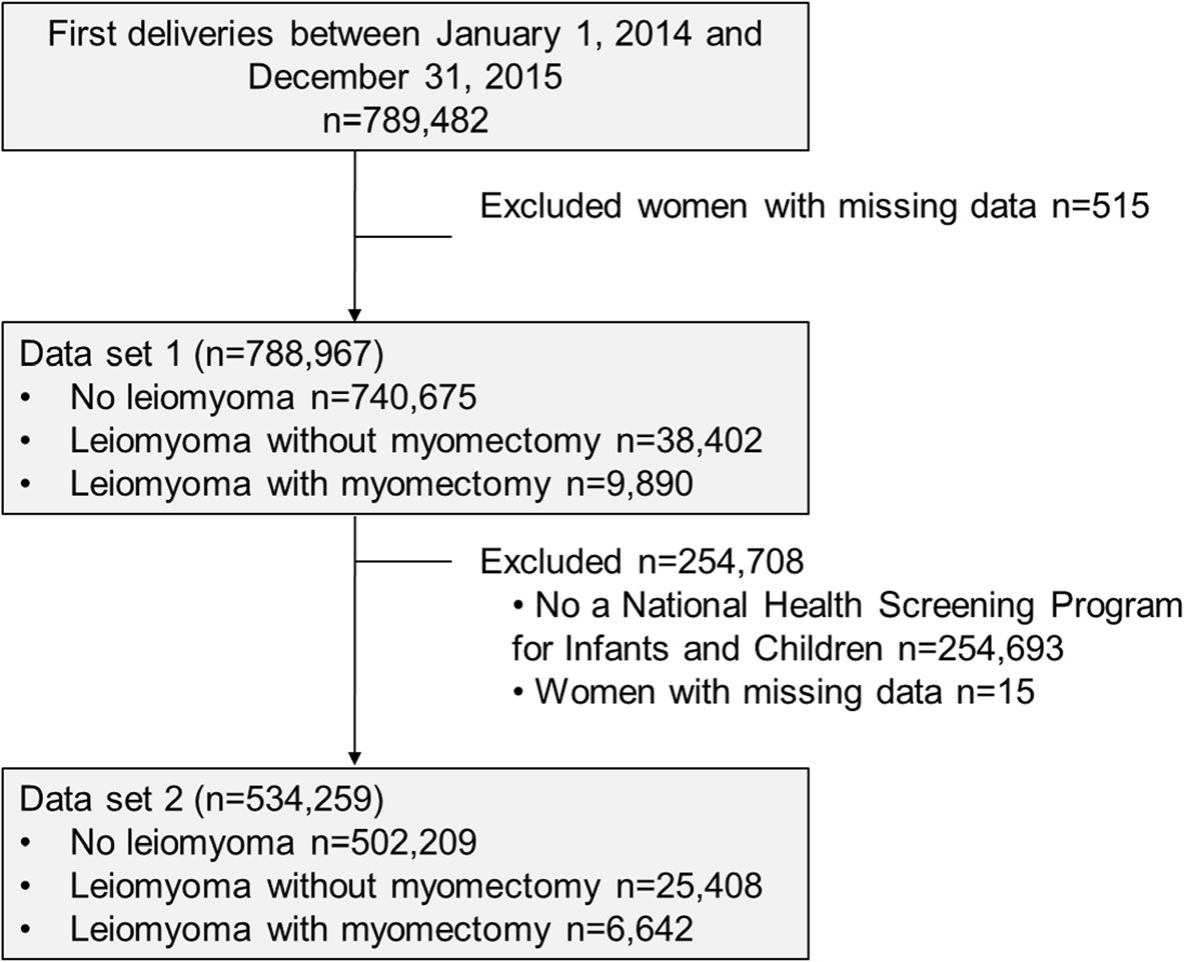
Flow chart of study participants’ enrollment

The flow chart of study participants’ enrollment is presented in Fig. 1. For pregnancy outcomes, we extracted information on delivery mode, nulliparity, multiple pregnancy, preeclampsia, PPH, placental abruption, placenta previa, and uterine rupture using the KNHI claims database (dataset 1). For the other outcomes, the KNHI claims database and the NHSP-IC database were merged. Because data on preterm birth and birth weight were corrected from the NHSP-IC database, women were excluded from analysis if their offspring did not undergo at least one of the seven consecutive NHSP-IC examinations or had missing data (dataset 2). Data on preterm birth, low birth weight (LBW), and large for gestational age (LGA) were extracted from dataset 2. Preterm birth was defined as gestational age at birth < 37 weeks. LBW and LGA were defined as birth weight < 2.5 kg and > 4.0 kg, respectively.

### Statistical analysis

Continuous and categorical variables are expressed as the mean ± standard deviation and percentages, respectively. Clinical characteristics were compared using the ANOVA for continuous variables and the chi-square test for categorical variables. Multivariate logistic regression analysis was used to estimate the adjusted odd ratio (aOR) and the 95% confidence interval (CI) s for the association of a presence of leiomyoma and myomectomy with adverse pregnancy outcomes. For multivariate analyses, a fixed set of known risk factors for adverse pregnancy outcomes was adjusted for potential confounding: maternal age (categorical variable; < 35 or ≥ 35 years old), and nulliparity (categorical variable). Statistical analyses were done using SAS for Windows v9.4 (SAS, Inc., Cary, NC).

## Results

From 2014 to 2015, 789,482 women delivered in Korea. After excluding women with missing data (*n* = 515), 788,967 remaining women were included in dataset 1. Among them, 740,675 (93.88%) women had no diagnosed myoma, 38,402 (4.88%) had diagnosed myoma(s) but no history of myomectomy, and 9890 (1.25%) women had a history of myomectomy before pregnancy. Dataset 2 [11] included 534,259 women, excluding women whose infants did not receive infant screening (*n* = 254,693) or showed missing values (*n* = 15). There were 502,209 (94.00%) women who had never been diagnosed with myoma, 25,408 (4.76%) women who had diagnosed myoma(s) but no history of myomectomy, and 6642 (1.24%) women who had a history of myomectomy, in dataset 2.

Maternal characteristics and adverse pregnancy outcomes of the study population in dataset 1 as described in Table 1. There were differences in maternal age, rates of nulliparity, multiple pregnancy, cesarean section, and complications of preeclampsia, postpartum hemorrhage, placenta previa, and uterine rupture, between the groups. However, there was no significant difference in placental abruption between the three groups.

**Table 1:** Demographics and birth outcomes of study population (Dataset 1)

	Group A^a^ (***N*** = 740,675)	Group B^a^ (***N*** = 38,402)	Group C^a^ (***N*** = 9890)	***p***-value
**Age**	31.62 ± 3.96	33.87 ± 3.50	34.63 ± 3.42	<.0001
**Nulliparity**	385,663 (52.07)	19,541 (50.89)	5380 (54.40)	<.0001
**Multiple pregnancy**	12,495 (1.69)	1074 (2.80)	432 (4.37)	<.0001
**Cesarean section**	286,946 (38.74)	17,547 (45.69)	8447 (85.41)	<.0001
**Preeclampsia**	16,033 (2.16)	1063 (2.77)	327 (3.31)	<.0001
**PPH**	72,501 (9.79)	3946 (10.28)	1007 (10.18)	0.0035
**Placental abruption**	2728 (0.37)	170 (0.44)	40 (0.40)	0.0572
**Placenta previa**	9365 (1.26)	848 (2.21)	289 (2.92)	<.0001
**Uterine rupture**	107 (0.01)	12 (0.03)	22 (0.22)	<.0001

^a^Group A, Women who had never been diagnosed with myoma; Group B, Women who had diagnosed myoma(s) but no history of myomectomy; Group C, Women who had a history of myomectomy

*PPH* Postpartum hemorrhage

Values are given as mean ± standard deviation or number (%)

Obstetric characteristics in dataset 2 are presented in Table 2. There were significant differences in neonatal birth weight and sex, and rates of preterm birth, LBW, and LGA.

**Table 2:** Demographics and birth outcomes of study population (Dataset 2)

	Group A^a^ (***N*** = 502,209)	Group B^a^ (***N*** = 25,408)	Group C^a^ (***N*** = 6642)	***p***-value
**Preterm birth**	13,470 (2.68)	1018 (4.01)	327 (4.92)	<.0001
**Neonatal sex–male**	258,518 (51.48)	13,066 (51.42)	3430 (51.64)	<.0001
**Birth weight (kg)**	3.20 ± 0.46	3.17 ± 0.50	3.11 ± 0.50	<.0001
**LBW**	20,203 (4.02)	1452 (5.71)	459 (6.91)	<.0001
**LGA**	17,390 (3.46)	885 (3.48)	165 (2.48)	<.0001

^a^Group A, Women who had never been diagnosed with myoma; Group B, Women who had diagnosed myoma(s) but no history of myomectomy; Group C, Women who had a history of myomectomy

*LBW* Low birth weight, *LGA* Large for gestational age

Values are given as mean ± standard deviation or number (%)

### Multivariate logistic regression analysis for adverse obstetric outcomes in pregnancies with myoma or following myomectomy

**Fig. 2:**
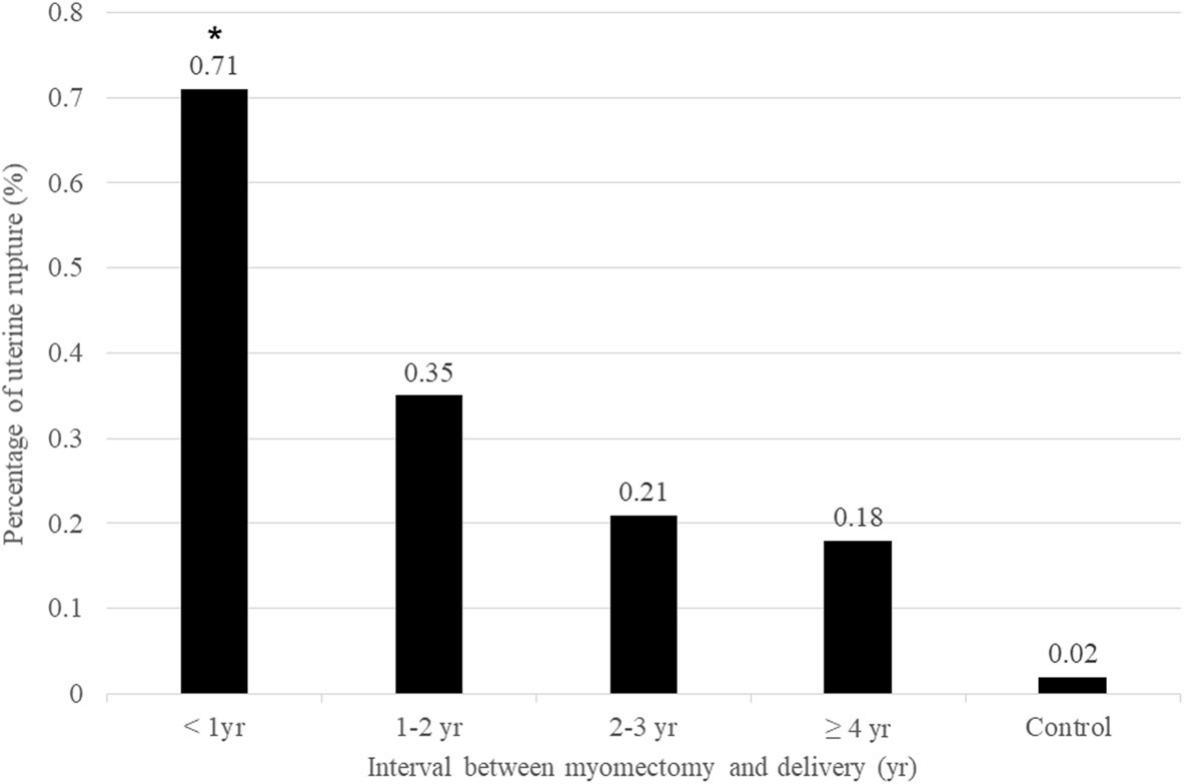
Incidence of uterine rupture in women with myomectomy, according to time interval after myomectomy

Both groups of women with a history of diagnosed myoma(s) and women with a history of myomectomy had significantly higher risks of cesarean section (aOR 1.13, 95% CI 1.1–1.16 and aOR 7.46, 95% CI 6.97–7.98, respectively) and placenta previa (aOR 1.41, 95% CI 1.29–1.54 and aOR 1.58, 95% CI 1.35–1.83, respectively), compared to women without a diagnosed myoma (Table 3). However, women with a history of myomectomy had a significantly higher risk of uterine rupture (aOR 12.78, 95% CI 6.5–25.13). We evaluated incidences of uterine rupture in women with myomectomy, according to the time interval after myomectomy (Fig. 2). The incidence of uterine rupture was significantly higher at delivery within one year after myomectomy (0.71%) compared to any longer delivery interval after myomectomy.

**Table 3:** The obstetric outcomes in women with diagnosed myoma(s) or a history of myomectomy (Dataset 1)

Obstetric outcomes	Odds ratio (95% confidence interval)	
	Unadjusted	Adjusted ^**b**^
**Cesarean section**		
**Group A**^a^	1	1
**Group B**^a^	1.312 (1.279–1.346)	1.193 (1.163–1.224)
**Group C**^a^	8.992 (8.408–9.617)	7.985 (7.463–8.544)
**Preeclampsia**		
**Group A**	1	1
**Group B**	1.326 (1.227–1.433)	1.124 (1.123–1.313)
**Group C**	1.360 (1.175–1.574)	1.159 (1.000–1.343)
**PPH**		
**Group A**	1	1
**Group B**	1.048 (1.005–1.092)	1.049 (1.006–1.094)
**Group C**	1.064 (0.983–1.152)	1.062 (0.981–1.151)
**Placental abruption**		
**Group A**	1	1
**Group B**	1.185 (0.970–1.448)	1.118 (0.914–1.368)
**Group C**	1.156 (0.784–1.704)	1.037 (0.703–1.532)
**Placenta previa**		
**Group A**	1	1
**Group B**	1.797 (1.646–1.962)	1.549 (1.418–1.692)
**Group C**	2.223 (1.912–2.584)	1.772 (1.523–2.063)
**Uterine rupture**		
**Group A**	1	1
**Group B**	2.421 (1.037–5.651)	2.032 (0.865–4.777)
**Group C**	17.00 (8.836–32.708)	13.674 (6.986–26.765)

^a^Group A, Women who had never been diagnosed with myoma; Group B, Women who had diagnosed myoma(s) but no history of myomectomy; Group C, Women who had a history of myomectomy

^b^Adjusted for age and parity

*PPH* Postpartum hemorrhage

We compared obstetric outcomes in dataset 2 (Table 4). Both groups of women with a history of diagnosed myoma or myomectomy had significantly higher risks of preterm birth and LBW than did women without a diagnosed myoma. However, women with diagnosed myoma(s) and women with a history of myomectomy had a significantly lower risk of LGA.

**Table 4:** The obstetric outcomes in women with diagnosed myoma(s) or a history of myomectomy (Dataset 2)

Obstetric outcomes	Odds ratio (95% confidence interval)	
	Unadjusted	Adjusted ^**b**^
**Preterm birth**		
**Group A**^a^	1	1
**Group B**^a^	1.514 (1.419–1.616)	1.426 (1.336–1.523)
**Group C**^a^	1.879 (1.679–2.103)	1.698 (1.517–1.902)
**LBW**		
**Group A**	1	1
**Group B**	1.446 (1.369–1.528)	1.355 (1.282–1.432)
**Group C**	1.771 (1.609–1.949)	1.580 (1.435–1.741)
**LGA**		
**Group A**	1	1
**Group B**	1.006 (0.939–1.078)	0.981 (0.916–1.051)
**Group C**	0.711 (0.609–0.830)	0.688 (0.589–0.803)

^a^Group A, Women who had never been diagnosed with myoma; Group B, Women who had diagnosed myoma(s) but no history of myomectomy; Group C, Women who had a history of myomectomy

^b^Adjusted for age and parity

*LBW* Low birth weight, *LGA* Large for gestational age

. When the myomectomy group was divided to two groups based on the types of myomas, the results were similar in women with submucosal or intramural myomas and subserosal or unspecified myomas (suppl 1&2).

### Multivariate logistic regression analysis for adverse obstetric outcomes in pregnancies with myoma or following myomectomy, in nulliparous women

In nulliparous women, both groups of women with a history of diagnosed myoma(s) and women with a history of myomectomy had significantly higher risks of cesarean section (aOR 1.13, 95% CI 1.1–1.17 and aOR 6.38, 95% CI 5.84–6.97, respectively), placenta previa (aOR 1.5, 95% CI 1.34–1.67 and aOR 1.78, 95% CI 1.49–2.13, respectively), and uterine rupture (aOR 4.14, 95% CI 1.14–15.06 and aOR 41.35, 95% CI 16.18–105.69, respectively), compared to women without a diagnosed myoma (Table 5). Risk of preeclampsia was slightly increased in women with a history of diagnosed myoma(s) (aOR 1.15, 95% CI 1.05–1.27), compared to women without a diagnosed myoma. In obstetric outcomes of dataset 2, among nulliparous women, both groups of women with a history of diagnosed myoma(s) and women with a history of myomectomy had significantly higher risks of preterm birth (aOR 1.45, 95% CI 1.33–1.57 and aOR 1.79, 95% CI 1.57–2.05, respectively), and LBW (aOR 1.31, 95% CI 1.23–1.41 and aOR 1.55, 95% CI 1.38–1.74, respectively), compared to women without a diagnosed myoma (Table 6). In nulliparous women, women with a history of myomectomy had a significantly lower risk of LGA (aOR 0.66, 95% CI 0.53–0.82), compared to women without a diagnosed myoma.

**Table 5:** The obstetric outcomes in nulliparous women with diagnosed myoma(s) or a history of myomectomy (Dataset 1)

Obstetric outcomes	Odds ratio (95% confidence interval)	
	Unadjusted	Adjusted ^**b**^
**Cesarean section**		
**Group A**^a^	1	1
**Group B**^a^	1.386(1.339–1.435)	1.210(1.168–1.253)
**Group C**^a^	8.194(7.502–8.949)	6.908(6.320–7.551)
**Preeclampsia**		
**Group A**	1	1
**Group B**	1.289(1.173–1.418)	1.180(1.072–1.298)
**Group C**	1.353(1.139–1.607)	1.180(0.992–1.403)
**PPH**		
**Group A**	1	1
**Group B**	1.045(0.988–1.105)	1.050(0.993–1.111)
**Group C**	1.037(0.934–1.151)	1.046(0.942–1.162)
**Placental abruption**		
**Group A**	1	1
**Group B**	1.113(0.863–1.435)	1.054(0.816–1.361)
**Group C**	1.015(0.619–1.664)	0.933(0.567–1.533)
**Placenta previa**		
**Group A**	1	1
**Group B**	2.008(1.798–2.242)	1.689(1.510–1.890)
**Group C**	2.672(2.236–3.193)	2.059(1.720–2.466)
**Uterine rupture**		
**Group A**	1	1
**Group B**	5.071(1.431–17.972)	4.667(1.295–16.816)
**Group C**	54.783(23.070–130.090)	48.206(19.239–120.789)

^a^Group A, Women who had never been diagnosed with myoma; Group B, Women who had diagnosed myoma(s) but no history of myomectomy; Group C, %) women who had a history of myomectomy

^b^Adjusted for age

*PPH* Postpartum hemorrhage

**Table 6:** The obstetric outcomes in nulliparous women with diagnosed myoma(s) or a history of myomectomy (Dataset 2)

Obstetric outcomes	Odds ratio (95% confidence interval)	
	Unadjusted	Adjusted ^**b**^
**Preterm birth**		
**Group A**^a^	1	1
**Group B**^a^	1.649(1.520–1.788)	1.510(1.390–1.639)
**Group C**^a^	2.145(1.876–2.452)	1.874(1.636–2.145)
**LBW**		
**Group A**	1	1
**Group B**	1.512(1.412–1.620)	1.377(1.285–1.476)
**Group C**	1.883(1.678–2.114)	1.631(1.451–1.833)
**LGA**		
**Group A**	1	1
**Group B**	1.030(0.937–1.132)	1.021(0.928–1.123)
**Group C**	0.676(0.546–0.838)	0.667(0.538–0.827)

^a^Group A, Women who had never been diagnosed with myoma; Group B, Women who had diagnosed myoma(s) but no history of myomectomy; Group C, %) women who had a history of myomectomy

*LBW* Low birth weight, *LGA* Large for gestational age

## Discussion

### Main findings

In this study, [1] Women who have had a myomectomy had higher risks of cesarean section, placenta previa, preterm birth, LBW, and uterine rupture, but a lower risk of LGA, compared to women without a history of diagnosed myoma.; [2] The incidence of uterine rupture was higher at delivery within one year after myomectomy (0.71%) than during any longer delivery interval after myomectomy.; [3] Women with a history of diagnosed myoma had higher risks of cesarean section and placenta previa, but no increased risks of preterm birth, LBW, or uterine rupture, compared to women without a history of diagnosed myoma.; [4] In nulliparous women, both groups of women with a history of diagnosed myoma(s) and women with a history of myomectomy had higher risks of cesarean section, placenta previa, uterine rupture, preterm birth, and LBW, compared to women without a diagnoses myoma.; [5] Especially, aORs for uterine rupture in women with diagnosed myoma and women who have had a myomectomy were 4.14 and 41.35, respectively, in nulliparous women.

### Interpretation

Previous studies have also reported increased adverse pregnancy outcomes, including abnormal placentation, such as placenta previa or placenta accreta [9, 12, 13], preterm delivery, cesarean delivery, uterine rupture, and postpartum bleeding, in women with a history of myomectomy [14–17]. The true incidence of uterine rupture during subsequent pregnancy following myomectomy is difficult to establish, because most of the studies have been cases, case series, or small retrospective cohort studies that do not account for the total number of pregnancies achieved after myomectomy and their consequent outcomes. The incidences of preterm birth and uterine rupture after myomectomy have been variously reported to range from 3.1 to 35% [18]and from 0.2 to 3.7%, respectively [18, 19]. The previous systematic review including all cohort studies with at least five cases demonstrated that the overall incidence of uterine rupture after myomectomy was 0.93% (0.45–1.92%) (*n* = 7/756); specifically, it was 0.47% (0.13–1.70%) (*n* = 2/426) in women undergoing a trial of labor after myomectomy, and 1.52% (0.65–3.51%) (*n* = 5/330) in women before the onset of labor [10]. However, the number of pregnancies and viable deliveries after prior myomectomy were 2367 and 1284, respectively, from a total of 23 studies. In our study, pregnancy outcomes were available for 9890 women with a history of myomectomy, which was the largest population. In the previous studies, although uterine rupture occurred at various gestation, it occurred more often before the onset of labor, with a high rate of fetal loss [10, 19]. In this study, the incidence of uterine rupture in women with a history of myomectomy was 0.22%, which is less than the reported incidence of uterine rupture (0.4–0.7%) in a trial of labor after cesarean section [20]. Possible reasons can be a missing diagnosis when uterine rupture or dehiscence was combined with placental abruption or antepartum/postpartum bleeding in the middle of pregnancy. However, in this study, women with a history of myomectomy had more than a 12-fold risk of uterine rupture over that of women without a diagnosed myoma. In nulliparous women, women with a history of diagnosed myoma(s) and women with a history of myomectomy had 4.14-fold and 41.35-fold higher risks of uterine rupture, compared to it of women without a diagnoses myoma. Therefore, counseling for myomectomy in women who desire a pregnancy in the future should discuss the risk of adverse pregnancy outcomes, especially uterine rupture during pregnancy, which can be associated with fetal loss.

In a previous comparison study about delivery outcomes between pregnancies following myomectomy and myoma-complicated pregnancies, the latter showed better outcomes, including fewer cesarean sections, preterm births, and less blood loss, than outcomes of pregnancies after myomectomy, which were similar to the results of this study [21]. A recent retrospective cohort study [22] revealed that women with a history of myomectomy were associated with increased risks of intraoperative transfusion, bowel injury, and a cesarean hysterectomy.

Previously, ACOG stated that myomectomy should be considered for a woman with uterine leiomyomas who has undergone several unsuccessful IVF cycles despite appropriate ovarian response and good-quality embryos [23]. SOGC, ASRM, and French guidelines also stated that intramural myomas may have a negative effect on fertility, but treating them does not improve fertility, and myomectomy is therefore indicated only for symptomatic myomas [5, 23, 24], They emphasized that information should be provided about the risk of uterine rupture during a future pregnancy, before planning a myomectomy in women who might become pregnant later on.

### Strengths and limitations

A limitation of this study was our lack of data on number, size, or type of myomas, type of closure after myomectomy, number of suture layers, and use of electrocauterization, which may have important clinical significance. However, we divided myomectomy group to submucosal or intramural myomas and subserosal or unspecified myomas, based on diagnostic codes. The results were not different according to types of myomas, although it is difficult to define the unspecified myomas. The second limitation was that there was no information on the type of myomectomy (laparoscopic, open, hysteroscopic, or robot-assisted) or type of conception (natural, OS, OS-IUI, or IVF). And data about gestational age at uterine rupture was not available. Lastly, this study did not have data if myomectomy or cesarean section was performed before the study period (2004–2015).

However, this study included the largest population in the group with a history of diagnosed myoma(s) with and without myomectomy. In addition, the nationwide design of the original database can provide more generalized outcomes in pregnancies with diagnosed myoma(s) and with previous myomectomy. To our knowledge, this is the first study about incidence of uterine rupture in women with myomectomy, according to delivery time interval after myomectomy. The incidence of uterine rupture was highest within one year after surgery, in this study. A previous study has reported that incision healing after a caesarean section took at least 6 months for the complete involution and recovery of uterine zonal anatomy by magnetic resonance imaging [25]. The other study also reported that risk of uterine rupture was 3.12 fold in pregnant women less than < 12 months since their last caesarean delivery, compared to it in women more than 24 months since their last caesarean delivery, with the odds of rupture appearing to plateau for intervals beyond 12 months [26]. Lastly, we had subgroup analysis for adverse obstetric outcomes in nulliparous women, because it can clearly eliminate the effects of previous cesarean section, which could be performed before the study period. In nulliparous women, both groups of women with a history of diagnosed myoma(s) and women with a history of myomectomy had higher risks of cesarean section, placenta previa, uterine rupture, preterm birth, and LBW compare to women without a diagnoses myoma. However, the risk of uterine rupture was much higher in women with a history of myomectomy. These results might be useful in counseling when a woman, who might become pregnant later on, is diagnosed with uterine myoma.

## Conclusion

When a woman is diagnosed as having myoma(s) before pregnancy, counseling should include information about the risks of pregnancies with myoma(s) and after myomectomy. Especially, if myomectomy is considered before pregnancy, a woman should be counseled that her risk of uterine rupture, during pregnancies after myomectomy, can be increased.

## Supplementary Information

**Additional file 1Supplementary 1.** The obstetric outcomes in women with diagnosed myoma(s) or a history of myomectomy (Dataset 1). **Supplementary 2.** The obstetric outcomes in women with diagnosed myoma(s) or a history of myomectomy (Dataset 2).

## Data Availability

All data generated or analysed during this study are included in this published article.

## Abbreviations

ASRM: American Society for Reproductive Medicine
SOGC: Society of Obstetricians and Gynaecologists of Canada
KNHI: Korea National Health Insurance
NHSP-IC: National Health Screening Program for Infants and Children
LBW: Low birth weight
LGA: Large for gestational age
ART: Assisted reproductive treatment
PPH: Postpartum hemorrhage
