# Supplementary material for: Nationwide population-based cohort study of adverse obstetric outcomes in pregnancies with myoma or following myomectomy: retrospective cohort study
Source: BMC Pregnancy Childbirth. 2020 Nov 23;20:716. doi: 10.1186/s12884-020-03406-9 (PMC7685654; doi:10.1186/s12884-020-03406-9)
Supplement: Supplementary file 1 — Additional file 1Supplementary 1. The obstetric outcomes in women with diagnosed myoma(s) or a history of myomectomy (Dataset 1). Supplementary 2. The obstetric outcomes in women with diagnosed myoma(s) or a history of myomectomy (Dataset 2). [file 12884_2020_3406_MOESM1_ESM.docx]

**Suppl 1.** The obstetric outcomes in women with diagnosed myoma(s) or a history of myomectomy (Dataset 1)

| **Obstetric outcomes** | **Odds ratio (95% confidence interval)** | |
| --- | --- | --- |
|  | **Unadjusted** | **Adjusted ^a^** |
| **Cesarean section** |  |  |
| **Group A** | 1 | 1 |
| **Group B** | 1.312 (1.279 – 1.346) | 1.193 (1.163 – 1.224) |
| **Group C1** | 10.184 (8.836 – 11.738) | 8.904 (7.720 – 10.269) |
| **Group C2** | 8.636 (7.991 – 9.334) | 7.670 (7.094 – 8.293) |
| **Preeclampsia** |  |  |
| **Group A** | 1 | 1 |
| **Group B** | 1.326 (1.227 – 1.433) | 1.214 (1.122 – 1.312) |
| **Group C1** | 1.183 (0.864 – 1.620) | 1.001 (0.730 – 1.372) |
| **Group C2** | 1.352 (1.139 – 1.605) | 1.143 (0.962 – 1.358) |
| **PPH** |  |  |
| **Group A** | 1 | 1 |
| **Group B** | 1.048 (1.005 – 1.092) | 1.049 (1.006 – 1.094) |
| **Group C1** | 1.089 (0.929 – 1.277) | 1.088 (0.928 – 1.276) |
| **Group C2** | 1.046 (0.953 – 1.149) | 1.043 (0.949 – 1.145) |
| **Placental abruption** |  |  |
| **Group A** | 1 | 1 |
| **Group B** | 1.185 (0.970 – 1.448) | 1.118 (0.914 – 1.368) |
| **Group C1** | 1.279 (0.608 – 2.691) | 1.142 (0.542 – 2.406) |
| **Group C2** | 1.161 (0.738 – 1.827) | 1.036 (0.658 – 1.632) |
| **Placenta previa** |  |  |
| **Group A** | 1 | 1 |
| **Group B** | 1.797 (1.646 – 1.962) | 1.549 (1.418 – 1.692) |
| **Group C1** | 2.453 (1.838 – 3.272) | 1.920 (1.438 – 2.565) |
| **Group C2** | 2.182 (1.828 – 2.606) | 1.743 (1.458 – 2.083) |
| **Uterine rupture** |  |  |
| **Group A** | 1 | 1 |
| **Group B** | 2.421 (1.037 – 5.651) | 2.034 (0.865 – 4.780) |
| **Group C1** | 19.075 (5.944 – 61.213) | 14.799 (4.553 – 48.098) |
| **Group C2** | 17.002 (8.048 – 35.919) | 13.948 (6.504 – 29.910) |

*Group A, Women who had never been diagnosed with myoma; Group B, Women who had diagnosed myoma(s) but no history of myomectomy; Group C1, Women who had a history of myomectomy on submucosal or intramural myomas; Groups C2, Women who had a history of myomectomy on subserosal or unspecified myomas

PPH, Postpartum hemorrhage

^a^ Adjusted for age and parity

**Suppl 2.** The obstetric outcomes in women with diagnosed myoma(s) or a history of myomectomy (Dataset 2)

| **Obstetric outcomes** | **Odds ratio (95% confidence interval)** | |
| --- | --- | --- |
|  | **Unadjusted** | **Adjusted ^a^** |
| **Preterm birth** |  |  |
| **Group A** | 1 | 1 |
| **Group B** | 1.514 (1.419 – 1.616) | 1.426 (1.335 – 1.522) |
| **Group C1** | 1.839 (1.464 – 2.310) | 1.650 (1.312 – 2.073) |
| **Group C2** | 1.814 (1.588 – 2.073) | 1.635 (1.430 – 1.870) |
| **LBW** |  |  |
| **Group A** | 1 | 1 |
| **Group B** | 1.446 (1.369 – 1.528) | 1.355 (1.282 – 1.432) |
| **Group C1** | 1.725 (1.419 – 2.096) | 1.527 (1.256 – 1.857) |
| **Group C2** | 1.738 (1.553 – 1.946) | 1.543 (1.378 – 1.729) |
| **LGA** |  |  |
| **Group A** | 1 | 1 |
| **Group B** | 1.006 (0.939 – 1.078) | 0.981 (0.916 – 1.051) |
| **Group C1** | 0.637 (0.458 – 0.886) | 0.613 (0.440 – 0.853) |
| **Group C2** | 0.711 (0.593 – 0.853) | 0.689 (0.575 – 0.827) |

*Group A, Women who had never been diagnosed with myoma; Group B, Women who had diagnosed myoma(s) but no history of myomectomy; Group C1, Women who had a history of myomectomy on submucosal or intramural myomas; Groups C2, Women who had a history of myomectomy on subserosal or unspecified myomas

LBW, Low birth weight; LGA, Large for gestational age

^a^ Adjusted for age and parity
